# Plant-Based Dietary Patterns for Human and Planetary Health

**DOI:** 10.3390/nu14081614

**Published:** 2022-04-13

**Authors:** Joshua Gibbs, Francesco P. Cappuccio

**Affiliations:** 1 WHO Collaborating Centre for Nutrition, Division of Health Sciences, Warwick Medical School, University of Warwick, Coventry CV4 7LA, UK; 2 Department of Medicine, University Hospitals Coventry & Warwickshire NHS Trust, Coventry CV2 2DX, UK

**Keywords:** plant-based diet, planetary health, human health, sustainability, chronic disease prevention

## Abstract

The coronavirus pandemic has acted as a reset on global economies, providing us with the opportunity to build back greener and ensure global warming does not surpass 1.5 °C. It is time for developed nations to commit to red meat reduction targets and shift to plant-based dietary patterns. Transitioning to plant-based diets (PBDs) has the potential to reduce diet-related land use by 76%, diet-related greenhouse gas emissions by 49%, eutrophication by 49%, and green and blue water use by 21% and 14%, respectively, whilst garnering substantial health co-benefits. An extensive body of data from prospective cohort studies and controlled trials supports the implementation of PBDs for obesity and chronic disease prevention. The consumption of diets high in fruits, vegetables, legumes, whole grains, nuts, fish, and unsaturated vegetable oils, and low in animal products, refined grains, and added sugars are associated with a lower risk of all-cause mortality. Meat appreciation, health concerns, convenience, and expense are prominent barriers to PBDs. Strategic policy action is required to overcome these barriers and promote the implementation of healthy and sustainable PBDs.

## 1. Introduction

There is scientific consensus that anthropogenic greenhouse gas (GHG) emissions influence global warming and climate change [1]. To limit the negative consequences of climate change, 196 parties have committed to keep the increase in global average temperature below 2 °C above pre-industrial levels and try to limit warming to 1.5 °C [2]. The coronavirus pandemic has acted as a reset on global economies providing us with the opportunity to build back greener and maximize our chances of meeting the 1.5 °C target [3]. For example, the government of the United Kingdom (UK) has laid out a ten-point plan for a green industrial revolution in which they commit to transforming the energy sector, ending the sale of petrol and diesel cars, decarbonising public transport, developing greener buildings, investing in carbon capture and storage, and protecting the natural environment [4]. Worryingly, they failed to address agriculture in their plans. Revolutionizing agricultural systems should arguably be a top priority considering food production is the single largest cause of global environmental change [5]. Current agricultural practices constitute up to 30% of global anthropogenic GHG emissions [6] and 70% of freshwater use [7], whilst occupying approximately 40% of Earth’s land [8]. Therefore, innovation within the agricultural sector has the potential to generate substantial sustainability gains.

A possible line of action, that is receiving ever-increasing interest, is to transition towards a plant-based food system. Plant-based foods have a significantly smaller footprint on the environment than animal-based foods. Even the least sustainable vegetables and cereals cause less environmental harm than the lowest impact meat and dairy products [9]. On top of the low environmental impact of plant-based diets (PBDs), they may provide additional benefits to human health. Unhealthy diets now represent the largest burden of disease globally, presenting a greater risk to morbidity, disability, and mortality than unsafe sex, alcohol, drug, and tobacco use combined [5]. Adopting plant-based food systems may allow countries to reduce their environmental footprints and tackle their obesity and diet-related non-communicable disease burdens simultaneously. A few reviews have covered the planetary and human health benefits associated with PBDs; however, since their publication, additional data of relevance have become available [10,11,12]. The aim of this review is to provide a concise summary of the planetary and human health benefits associated with PBDs using evidence from the latest advances in the field. This review will also summarise the main barriers to PBDs and offer potential solutions.

PBD is an umbrella term that describes any dietary pattern that emphasises the consumption of foods derived from plants and excludes or limits the consumption of most or all animal products. PBDs can be healthy or unhealthy depending on their composition. Healthy PBDs focus on unprocessed plant foods, including fruits, vegetables, whole grains, legumes, nuts, and seeds, whereas unhealthy PBDs contain high quantities of processed and ultra-processed plant foods such as sugar-sweetened beverages, refined grains, sweets, and desserts. Descriptions of the various PBDs mentioned in this review are shown in Table 1.

## 2. Planetary Health

### 2.1. Greenhouse Gas (GHG) Emission

**Figure 1:**
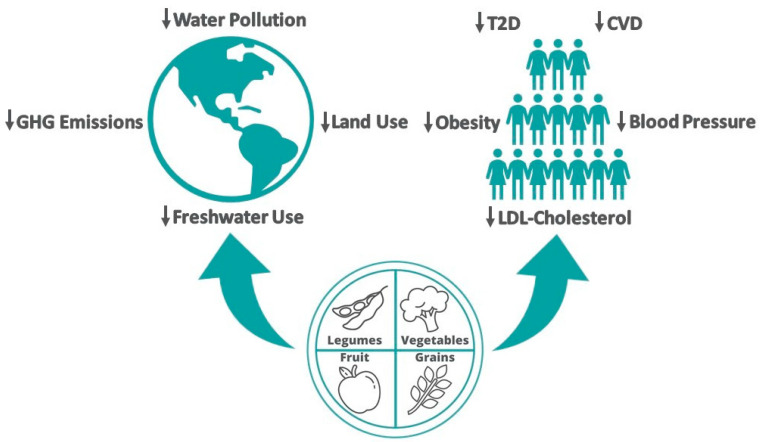
Summary of the planetary and human health benefits associated with the adoption of plant-based dietary patterns. Abbreviations: CVD, cardiovascular disease; GHG, greenhouse gas; LDL, low-density lipoprotein; T2D, type 2 diabetes.

Food systems are responsible for 21–37% of all GHG emissions globally [13]. Innovation and transformation within the food and agricultural sectors are imperative to limiting global warming to 1.5 °C. Between 2017 and 2018, agricultural emissions rose by 1.5% reaching a total of 5.6 GtCO_2_, even with modest improvements in efficiency [14]. Of this total, 52% was caused by cattle products, primarily meat and dairy. Per-capita emissions from food consumption are 39% and 41% higher in very high human development index (HDI) countries than in high HDI countries and low HDI countries, respectively [14]. These differences in emissions are despite the use of high emission-intensity beef farming in low HDI countries. In very high HDI countries, cattle products are responsible for 68% of total consumption-based agricultural GHG emissions [14]. Reducing red meat consumption is a major key to meeting emission targets for very high HDI countries and it would deliver substantial health co-benefits. The rate of red meat-related mortality is nearly nine times greater in very high HDI countries than in low HDI countries [14]. Life cycle assessment studies have shown that pork, chicken, and seafood produce less GHG emissions than beef; however, even the lowest impact animal products exceed the average GHG emissions of substitute plant proteins [9,15]. Moving to diets that exclude animal products could reduce global GHG emissions by 49% (Figure 1) [9].

### 2.2. Agricultural Land Use

Around 43% of the planet’s ice-free terrestrial landmass is occupied by farmland (including croplands and pasturelands). Approximately 83% of this farmland is used to produce meat, eggs, farmed fish, and dairy, yet they only provide 18% and 37% of our calories and protein, respectively [9]. Per kilogram, animal products require more lifecycle energy inputs than plant foods [16]. The adoption of PBDs would substantially reduce agricultural land use. Eshel et al. [17] estimated that Americans could save approximately 34% and 24% of dietary and total land use, respectively, if they replaced all meat with plant-based alternatives. Considering the amount of land required to produce animal products, it is unsurprising that they are accountable for 67% of the deforestation caused by agriculture [9]. The destruction of ecosystems for croplands and pasturelands is the single largest factor causing species to be threatened with extinction [18]. Biodiversity is essential for the productivity and resilience of our food systems [19]. Shifting to PBDs would slow biodiversity loss substantially, thus having a protective effect on global food security [5]. It is estimated that animal product-free diets have the potential to reduce diet-related land use by 3.1 billion hectares (76% reduction), including a 19% reduction in arable land (Figure 1) [9].

### 2.3. Water Use

In total, 70% of all global freshwater withdrawals are used for the irrigation of crops, of which 24% are fed to livestock [5,20]. Approximately 43,000 L of water are required to produce 1 kg of beef, whereas it only takes 1000 L to produce 1 kg of grain [21]. A modelling study found that reducing animal product consumption would reduce global green and blue water use by 21% and 14%, respectively (Figure 1) [22]. PBDs may therefore play a role in water conservation. Animal product-free diets may also improve water quality by reducing eutrophication caused by nitrogenous fertilizer and manure runoff by 49% (Figure 1) [9].

### 2.4. Healthy Reference Dietary Pattern

The EAT-Lancet Commission has developed a healthy reference dietary pattern that would allow humanity to stay within a safe operating space, in terms of climate change, land use, biodiversity loss, freshwater use, and nitrogen and phosphorus pollution, even with a 10 billion global population [5]. The dietary pattern largely consists of fruits and vegetables, whole grains, legumes, nuts, and unsaturated oils; low to moderate consumption of seafood and poultry; zero to low consumption of red meat, processed meat, added sugar, refined grains, and starchy vegetables. Using data from the European Prospective Investigation into Cancer and Nutrition (EPIC) cohort involving 443,991 participants, Laine et al. [23] estimated that up to 19–63% of deaths and up to 10–39% of cancers could be prevented in a 20-year risk period by adopting different levels of adherence to the EAT-Lancet reference diet. They also estimated that switching from low adherence to higher adherence could reduce food-associated greenhouse gas emissions by up to 50% and land use up to 62%. 

## 3. Human Health

Globally we are experiencing an unprecedented level of diet-related disease. Worldwide, 2.1 billion adults are overweight or obese [5]. Overweight and obesity are associated with a range of chronic diseases including type 2 diabetes (T2D) [24], hypertension [25], cardiovascular disease (CVD) [26], and some types of cancer [27]. Together, these diseases have a massive cost on society in terms of lives lost and healthcare spending. The Global Burden of Disease study estimated that increased consumption of whole grains, vegetables, nuts and seeds, and fruit could prevent 1.7 million, 1.8 million, 2.5 million, and 4.9 million premature deaths per year, respectively, via the beneficial effects on chronic disease risk factors [28].

### 3.1. Obesity

An extensive body of population studies and clinical trials supports the implementation of PBDs for the prevention of obesity and obesity-related diseases. Observational data from the Adventist Health Study-2 (AHS-2) involving 41,387 participants, showed that body mass index (BMI) was positively correlated with the amount of animal-based foods consumed, such that non-vegetarians had the highest BMI, followed by semi-vegetarians, pescatarians, vegetarians, and vegans [29]. In addition, findings from the EPIC-Oxford cohort, containing 21,966 men and women, have shown that vegans and pescatarian women gain significantly less weight annually compared with meat-eaters [30]. The lowest mean annual weight gain was observed in individuals who converted, during follow-up, to diets containing fewer animal-derived foods. In accordance with these findings, the European Prospective Investigation into Cancer, Physical Activity, Nutrition, Alcohol, Cessation of smoking, Eating out of home and obesity (EPIC-PANACEA) study found total meat consumption was positively associated with weight gain in 103,455 men and 270,348 women [31]. After adjusting for estimated energy intake, an additional 250 g/d of meat led to a 2 kg higher weight gain after 5 years (95% CI: 1.5, 2.7 kg). In a 5-year longitudinal study of 787 non-obese participants, dietary patterns were evaluated with overall plant-based diet index (PDI) scores, in which plant foods received positive scores and animal-derived foods received reverse scores [32]. A healthy PDI (hPDI) and an unhealthy PDI (uPDI) were also created. For the hPDI, healthy plant foods (fruits, vegetables, legumes, whole grains, nuts, and unsaturated vegetable oils) received positive scores, and animal foods and unhealthy plant foods (fruit juices, refined grains, and added sugars) received reverse scores. For the uPDI, unhealthy plant foods were allocated positive scores and animal foods and healthy plant foods were allocated reverse scores. At follow-up, both the hPDI (Risk Ratio (RR) = 0.31; 95% CI: 0.12–0.77) and overall PDI (RR = 0.56; 95% CI: 0.23–1.33) were inversely associated with obesity risk. However, only the hPDI association achieved statistical significance. Conversely, the uPDI was positively associated with obesity risk (RR = 1.94; 95% CI: 0.81–4.66); however, this finding was not statistically significant. 

Robust evidence from clinical trials supports the use of PBDs for weight loss. In 2015, Barnard et al. [33] performed a meta-analysis of 15 clinical trials with vegan and vegetarian interventions lasting four weeks or more with no energy restrictions. Consumption of PBDs was associated with a mean weight change of −3.4 kg (95% CI: −4.4, −2.4 kg) in an intention-to-treat analysis and −4.6 kg (−5.4, −3.8 kg) in a completer analysis (Figure 1). Similarly, a 2021 meta-analysis of seven clinical trials found that PBDs significantly lowered bodyweight in Type 2 diabetics (−2.35 kg (95% CI: −3.51, −1.19)) [34]. A few new clinical trials assessing the effect of PBDs on bodyweight have been published since 2015 [35,36,37,38,39,40]. The BROAD study, which prescribed a whole food PBD, had noteworthy results [38]. It showed greater weight loss at 6 and 12 months than any other comparable interventional trial (no energy restrictions or regular exercise orders) to date.

### 3.2. Type 2 Diabetes

The global prevalence of T2D has nearly doubled in the past 30 years [41]. In 2021, diabetes was responsible for 6.7 million deaths and $966 billion USD in health expenditure [42]. Large cohort studies show that the prevalence and incidence of T2D are significantly lower among those following PBDs. T2D prevalence in the AHS-2 cohort followed a similar trend as BMI with the lowest prevalence occurring in vegans (2.9%) and the highest in non-vegetarians (7.6%) [43]. Pescatarians (4.8%), semi-vegetarians (6.1%), and vegetarians (3.2%) had intermediate T2D prevalence. After adjusting for BMI and other confounding variables, vegans had half the risk of T2D than non-vegetarians (Odds Ratio (OR)) 0.51 (95% CI: 0.40, 0.66)) and semi-vegetarians had an intermediate risk (0.76 (0.65, 0.90)). In a 2-year prospective study of the AHS-2 cohort, vegans had less than half the risk of T2D than non-vegetarians (OR 0.38 (0.24, 0.62)) even when adjustments were made for BMI and other confounders [29]. In a 17-year prospective study with 8401 participants, long-term weekly dietary inclusion of meat was associated with 74% increased (OR 1.74 (1.36, 2.22)) odds of T2D compared with long-term adherence to a vegetarian dietary pattern [44]. Weekly meat intake remained an important risk factor (1.38 (1.06,1.68)) after adjusting for weight and weight change. 

In a prospective study of three US cohorts (Nurses’ Health Study (NHS), NHS II, Health Professionals Follow-up Study) totalling 192,657 participants, Chen et al. [45] evaluated the associations between changes in PBDs and subsequent T2D risk. During the 2,955,350 person-years of follow-up, 12,627 cases of T2D developed. Participant dietary patterns were evaluated with overall PDI, hPDI, and uPDI scores. Compared with participants whose indices remained stable over the 4-year follow-up, participants with the largest decrease (>10%) in PDI and hPDI had a 12–23% higher T2D risk in the subsequent 4 years. Each 10% increment in PDI and hPDI over 4 years was associated with a 7–9% lower T2D risk. It is worth noting that changes in the PDI scores were primarily due to changes in healthy plant-food intake, not changes in animal-derived food intake. No associations were observed between changes in uPDI and subsequent T2D risk. This may be due to the benefits of low animal food intake cancelling out the harmful effects associated with low intake of healthy plant foods [45].

A 2019 meta-analysis of nine prospective studies totalling 307,099 participants, found a significant inverse association between higher adherence to PBDs and T2D risk (RR 0.77 (95% CI: 0.71, 0.84)) in comparison with poorer adherence (Figure 1) [46]. As well as preventing T2D, there is evidence that PBDs may be an effective tool in the treatment of the disease. A meta-analysis of six controlled clinical trials found that consumption of PBDs was associated with a significant reduction in haemoglobin A1c (−0.39 points) compared with the consumption of omnivorous control diets [47]. This hypoglycaemic effect is approximately half of that observed with the prescription of the first-line medication, metformin [48]. 

### 3.3. CVD Risk

CVDs are the leading cause of mortality globally. In 2019, CVDs were responsible for 18.6 million deaths worldwide [49]. There is a range of evidence that supports the use of PBDs for the prevention of CVDs. A 2021 meta-analysis of prospective cohort studies totalling 698,707 participants, found that PBDs were associated with a 16% lower risk of CVD and an 11% lower risk of coronary heart disease (CHD) [50]. However, there were no associations between PBDs and risk of stroke. Another 2021 meta-analysis of prospective cohort studies totalling 410,085 participants found that PBDs were associated with a 10% lower risk of CVD incidence and 8% lower risk of cardiovascular mortality [51]. In a randomised secondary prevention trial (The Lyon Diet Heart Study) with 275 events recorded during a mean follow-up of 46 months, adherence to a plant-based Mediterranean-type dietary pattern was associated with a 72% reduction in cardiovascular events compared with adherence to a western-type dietary pattern [52]. In a randomised controlled trial with a 5-year follow-up, intensive lifestyle changes including the adoption of a healthful plant-based dietary pattern were shown to cause regression of atherosclerosis [53]. The control group in this trial had more than twice the risk of a cardiovascular event than the intensive lifestyle changes group (Figure 1). The reduced risk of CVD incidence and cardiovascular mortality observed in those following PBDs is likely due to the beneficial effects on CVD risk factors including overweight or obesity, T2D, hypertension, and hypercholesterolemia.

### 3.4. Hypertension and Hypercholesterolemia

In the AHS-2 cohort, vegans had approximately half the odds of hypertension than omnivores, even after controlling for BMI [54]. A 2021 meta-analysis including 41 controlled trials and 8416 participants found that PBDs significantly lower both systolic and diastolic blood pressure even with the inclusion of some animal products (Figure 1) [55]. A 2017 meta-analysis of 19 clinical trials including 1484 participants, found that compared with the consumption of omnivorous diets, vegetarian diets were significantly associated with decreased total cholesterol (−12.5 mg/dL) and low-density lipoprotein cholesterol (−12.2 mg/dL) (Figure 1) [56].

### 3.5. CVD Prevention

Taken together, the beneficial effects of PBDs on chronic disease risk factors found in controlled trials, and their associations with lower chronic disease risk found in prospective cohort studies provide strong support for the implementation of PBDs for chronic disease prevention. In a prospective cohort of 315,919 participants, high hPDI scores were associated with a 36% lower risk of mortality and each 10-point increase was associated with a 19% lower risk [57]. On the other hand, high uPDI scores were associated with a 41% increase in mortality risk and each 10-point increase was associated with a 15% increase in risk. This is supported by the most comprehensive systematic review on dietary patterns and all-cause mortality (ACM) to date [58]. It found that dietary patterns characterised by higher intake of vegetables, legumes, fruits, nuts, unrefined grains, fish, and unsaturated vegetable oils, and lower or no consumption of animal products (red and processed meat, meat and meat products, and high-fat dairy), refined grains, and added sugar, were associated with lower ACM risk.

## 4. Barriers and Potential Solutions

**Figure 2:**
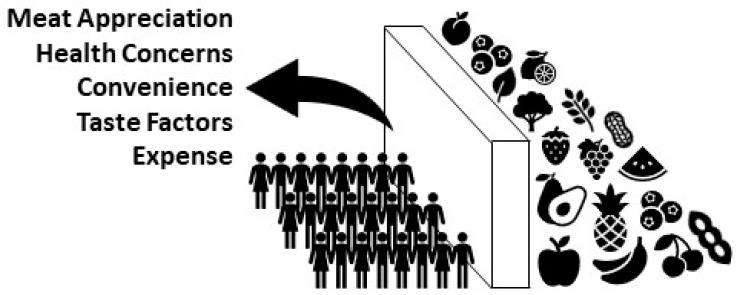
The main barriers to widespread adoption of plant-based dietary patterns.

In 2020, a comprehensive review of the literature outlined the most prominent perceived and objective barriers preventing people from switching to PBDs [59]. The most prominent barrier to PBDs is meat appreciation and the difficulty perceived in abstaining from consumption (Figure 2). The development of plant-based meat alternatives provides an opportunity to overcome this barrier. Plant-based products have been developed to visually resemble meat and match the taste, structure, and nutritional value preferences of meat eaters. These products make the transition to PBDs less difficult and more appealing. Environmental life cycle assessments for two popular plant-based substitutes, Beyond Meat’s Beyond Burger and Impossible Food’s Impossible Burger, showed that switching from beef to either of the products reduces GHG emissions, land use, and water footprint by approximately 90% [60,61]. Although plant-based meat alternatives are classified as ultra-processed, they may still exert some of the beneficial effects on CVD risk factors as healthy PBDs [62]. A randomized cross-over trial investigating the effect of Beyond Meat products versus animal-derived meat on CVD risk factors found that consumption of plant-based meat alternatives was associated with significantly lower trimethylamine-N-oxide (TMAO) concentrations, LDL-cholesterol concentrations, and body weight compared with the consumption of animal meat [63]. Moreover, there were no adverse effects on other risk factors during the plant-based phase. More controlled trials are needed to characterize the effect of ultra-processed meat analogues on health markers. 

The second most prominent barrier to PBDs is health concerns, specifically nutrient deficiencies, for example, protein and calcium (Figure 2) [59]. International and national commitments to PBDs demonstrated by investment in public health and sustainability education could break down these barriers. The public needs to be educated on specific plant-based food sources of essential nutrients such as iron, calcium, and zinc and be reassured that their protein needs can be sufficiently met. A potential strategy for relieving the perceived health concerns attached to PBDs is to provide proper nutrition education to medical students and health professionals. A survey of medical schools found that on average fewer than 20 h over four years are spent on nutrition education [64]. Accordingly, physicians often lack important nutrition knowledge and the counselling skills required to successfully guide their patients [65,66,67,68,69,70,71,72,73,74,75]. In a survey of resident physicians, only 14% of participants felt physicians were adequately trained to provide nutritional counselling [76]. Ironically, in a survey of the public, 61% of participants considered physicians to be “very credible” sources of nutrition information [77]. Educating doctors on how to prevent and treat chronic diseases with healthful PBDs may have positive effects beyond individual patient care, by influencing the wider public’s negative perceptions of PBDs. However, a lack of nutrition training is not the only way that physicians act as barriers. Firstly, they may have conflicts of interest and personal prejudices that bias their views on PBDs, preventing them from promoting the implementation of PBDs. Secondly, there is a lack of financial incentive for physicians to implement the use of PBDs [78,79]. Preventing chronic diseases with healthful PBDs reduces the demand for expensive medical treatments and procedures, which results in reduced income for physicians. 

The third most common barrier relates to convenience and tastes factors (Figure 2) [59]. The availability of plant-based options out of home are limited and people believe that the preparation of plant-based meals is complicated. PBDs are also perceived as tasteless [80]. New policies mandating that canteens at schools, hospitals, universities, and other state-owned services must provide healthful plant-based options could be implemented to reduce the convenience barrier. Incentives for businesses to offer more healthful plant-based options would also help to overcome this barrier. Online educational resources and community cooking classes could be utilized to facilitate the teaching of plant-based food preparation to the public, potentially tacking both convenience and taste factors [79]. Taste barriers could also be overcome with the previously mentioned meat analogues.

The final prominent barrier to PBDs is the expense of plant-based foods (Figure 2) [59]. This barrier could be broken down by allocating subsidies to the production of sustainable, healthful foods (e.g., fruits and vegetables) financed by a tax on unhealthful, environmentally damaging foods (e.g., red and processed meat) or an incremental increase in income tax [81]. It is estimated that a subsidy of 25% of the cost of fruits and vegetables could close the gap between the recommended intake and the actual average intake by a third [81]. 

## Figures and Tables

**Table 1:** Descriptions of various plant-based dietary patterns.

Dietary Pattern	Description
Healthful plant-based	High consumption of fruits, vegetables, legumes, whole grains, nuts, and unsaturated vegetable oils, and lower or no consumption of animal products (meat, fish, poultry, dairy, and eggs) and processed foods
Unhealthful plant-based	High consumption of fruit juices, sugar-sweetened beverages, refined grains, potatoes, and sweets and desserts, and lower consumption of animal products (meat, fish, poultry, dairy, and eggs) and healthy plant foods (fruits, vegetables, legumes, whole grains, nuts, and unsaturated vegetable oils).
Vegan	Excludes all animal products (meat, fish, poultry, dairy, and eggs) and is based solely on plant-based foods
Vegetarian	Excludes meat, fish, and poultry but does include eggs and dairy, in addition to plant-based foods
Pescatarian	Excludes meat and poultry but includes fish, dairy, and eggs, in addition to plant-based foods
Semi-vegetarian	Includes all animal products, including meat, fish, poultry, dairy, and eggs, in addition to plant-based foods. However, red meat intake is limited
EAT-Lancet reference	Consists of fruits and vegetables, whole grains, legumes, nuts, and unsaturated oils; low to moderate consumption of seafood and poultry; zero to low consumption of red meat, processed meat, added sugar, refined grains, and starchy vegetables

## Data Availability

Not applicable.
